# Leaves play a central role in the adaptation of nitrogen and sulfur metabolism to ammonium nutrition in oilseed rape (*Brassica napus*)

**DOI:** 10.1186/s12870-017-1100-9

**Published:** 2017-09-20

**Authors:** Inmaculada Coleto, Marlon de la Peña, Jon Rodríguez-Escalante, Iraide Bejarano, Gaëtan Glauser, Pedro M. Aparicio-Tejo, M. Begoña González-Moro, Daniel Marino

**Affiliations:** 1 Department of Plant Biology and Ecology, University of the Basque Country (UPV/EHU), Apdo. 644, E-48080 Bilbao, Spain; 2 Neuchâtel Platform of Analytical Chemistry, University of Neuchâtel, Avenue de Bellevaux 51, 2000 Neuchâtel, Switzerland; 3 Departamento de Ciencias del Medio Natural, Universidad Pública de Navarra, Pamplona, Navarre Spain; 4 Ikerbasque, Basque Foundation for Science, E-48011 Bilbao, Spain

**Keywords:** Ammonium, *Brassica napus*, Glucosinolates, Nitrate, Nitrogen, Oilseed rape, Sulfur

## Abstract

**Background:**

The coordination between nitrogen (N) and sulfur (S) assimilation is required to suitably provide plants with organic compounds essential for their development and growth. The N source induces the adaptation of many metabolic processes in plants; however, there is scarce information about the influence that it may exert on the functioning of S metabolism. The aim of this work was to provide an overview of N and S metabolism in oilseed rape (*Brassica napus*) when exposed to different N sources. To do so, plants were grown in hydroponic conditions with nitrate or ammonium as N source at two concentrations (0.5 and 1 mM).

**Results:**

Metabolic changes mainly occurred in leaves, where ammonium caused the up-regulation of enzymes involved in the primary assimilation of N and a general increase in the concentration of N-compounds (NH_4_^+^, amino acids and proteins). Similarly, the activity of key enzymes of primary S assimilation and the content of S-compounds (glutathione and glucosinolates) were also higher in leaves of ammonium-fed plants. Interestingly, sulfate level was lower in leaves of ammonium-fed plants, which was accompanied by the down-regulation of *SULTR1* transporters gene expression**.**

**Conclusions:**

The results highlight the impact of the N source on different steps of N and S metabolism in oilseed rape, notably inducing N and S assimilation in leaves, and put forward the potential of N source management to modulate the synthesis of compounds with biotechnological interest, such as glucosinolates.

**Electronic supplementary material:**

The online version of this article (10.1186/s12870-017-1100-9) contains supplementary material, which is available to authorized users.

## Background

Nitrogen (N) and sulfur (S) are major plant nutrients that serve as constituents of proteins and several other important organic compounds. A close interaction between N and S in terms of uptake, reduction and assimilation has been widely reported in the literature [1, 2]. N is mainly taken up from soil in the form of nitrate (NO_3_^−^) and ammonium (NH_4_^+^/NH_3_). The source of N affects many metabolic and physiological processes and may even influence plant adaptation to the environment [3]. In general, the preference of plants towards NO_3_^−^ or NH_4_^+^ depends both on the genotype and on environmental variables such as soil pH and the availability of other nutrients [4]. However, most crops show stress symptoms, generally manifested as growth reduction, in presence of moderate to high NH_4_^+^ concentrations. The reason of the negative effect of NH_4_^+^ on plant growth is not completely clear; in general, it is considered that its rapid uptake and excessive accumulation in tissues are the main causes of the toxicity symptoms [4]. Indeed, a common response of plants growing with NH_4_^+^ is the induction of the N primary assimilatory machinery to control NH_4_^+^ homeostasis [5, 6].

N availability has been shown for many years to disrupt the metabolism of S, and vice versa. Indeed, sulfate (SO_4_^2−^) flux inside the root has been reported to be regulated by N availability [7, 8]. Moreover, N deprivation has been shown to provoke the down-regulation of SO_4_^2−^ transporters and the addition of reduced N to stimulate their expression [9, 10]. Once in the cell, SO_4_^2−^ is reduced and incorporated as sulfide into the amino acid skeleton *O*-acetylserine to form cysteine (Cys). Then, Cys can act as sulfur donor for the synthesis of the amino acid methionine (Met). The correct supply of *O*-acetylserine, essential for Cys biosynthesis, is dependent on serine and thus, again on adequate N availability [1, 11]. Furthermore, N deprivation has been shown to down-regulate the expression and the activity of S-assimilating enzymes while N supply stimulates S incorporation into biomolecules [1, 12].

Glutathione (GSH) is one of the major organic S-containing compounds transported and stored in plants. Among its multiple roles, it acts as regulator of cellular S homeostasis and controls cell redox status, being one of the most important plant antioxidants [13]. Its biosynthesis depends on the availability of its precursors, the amino acids Cys, glutamic acid and glycine, and therefore, on the availability of N and S [14]. In the Brassicaceae family, other abundant S-containing compounds are glucosinolates (GLS), a large group of secondary metabolites whose common structure comprises a β-D-thioglucose group, a sulfonated oxime moiety and a variable side-chain derived from Met, tryptophan (Trp) or phenylalanine (Phe) [15]. Depending on the structure of their amino acid precursor, GLS are classified into three groups: aliphatic (mainly derived from Met), indolic (derived from Trp) and aromatic (derived from Phe) [15]. The degradation products of GLS are bioactive defence molecules that protect cruciferous plants against insects, herbivores and certain microbial pathogens [16]. Furthermore, they are responsible for the characteristic smell and taste of cruciferous vegetables [17]. In recent years, considerable attention has been paid to GLS because of their potential anticancer activity [18]. In particular, sulforaphane, an isothiocyanate derived from the hydrolysis of the aliphatic GLS glucoraphanin, is considered one of the most potent naturally occurring inducers of enzymes that reduce DNA damage [19]. However, the ingestion of large amount of GLS is thought to be deleterious to domestic livestock health and production [20]. Therefore, the positive and negative effects of GLS on the quality of both human and animal foods and their role in the defence against crop pests have increased the interest regarding the possibility of manipulating GLS levels to produce new and improved commercial cruciferous crop varieties [18].

A number of studies have analyzed the influence of N nutrition on GLS content in tissues of different Brassicaceae species. In general, GLS content raises when increasing N fertilization; however, since both amino acid precursors and a sulfur donor are necessary for GLS synthesis, a proper balance between N and S metabolism is required [21, 22]. Few studies have paid attention to the regulation of GLS synthesis in plants grown with NH_4_^+^ as N source. Some studies reported no differences or even reduced levels of GLS in plants cultured with NH_4_^+^ as sole N source [23, 24], while other showed increased GLS levels in response to NH_4_^+^ availability [25, 26]. Recently, we also reported the activation of GLS metabolism in leaves of *A. thaliana* and broccoli plants grown under ammonium nutrition suggesting N management as a way to control GLS metabolism [27].

The present work aims to provide an integrative view on how the exclusive access to a different N source influences N assimilation and its relationship with S metabolism in oilseed rape. To do so, we grew plants hydroponically with exclusive nitrate or ammonium supply (0.5 or 1 mM), examined the whole plant physiological status and determined different parameters of N and S metabolism in leaf and root. The overall results evidence a strong interaction between N and S metabolism and put forward the relevance of considering N source to manage sulfur usage in oilseed rape, which will ultimately determine the concentration of S-compounds such as glucosinolates.

## Methods

### Growth conditions and experimental design

Seeds of *Brassica napus* cv. ES Neptune (Euralis Semillas S.A.) were sterilized by immersion in 70% ethanol for 30 s, then in 1% bleach for 7 min and finally rinsed 5 times with deionised water. Seeds were sown in trays filled with perlite-vermiculite 1:1 (*v*/v) inert substrate mixture and watered with deionised water. Trays were kept during 4 days in the dark at 4 °C for vernalization and transferred into a phytotron [60/70% of humidity, 23 °C day (14 h) with a light intensity of 400 μmol m^−2^ s^−1^ and 18 °C night (10 h)].

Seven day-old seedlings were washed with deionised water to remove traces of perlite-vermiculite and transferred to 5 L hydroponic tanks (10 plants per tank; for Additional file 1: Fig. S1 20 plants per tank). The nutrient solution contained 1.15 mM K_2_HPO_4_, 0.85 mM MgSO_4_, 0.7 mM CaSO_4_, 2.68 mM KCl, 0.5 mM CaCO_3_, 0.07 mM NaFeEDTA, 16.5 μM Na_2_MoO_4_, 3.7 μM FeCl_3_, 3.5 μM ZnSO_4_, 16.2 μM H_3_BO_3_, 0.47 μM MnSO_4_, 0.12 μM CuSO_4_, 0.21 μM AlCl_3_, 0.126 μM NiCl_2_ and 0.06 μM KI, pH 6.8. Two concentrations of N were tested for each source: 0.5 and 1 mM. To properly compare both N sources within each concentration studied, NO_3_^−^-fed plants were supplied with CaSO_4_ to equilibrate the SO_4_^−2^ supplied together with the NH_4_^+^. Thus, for 0.5 mM N treatment we applied 0.25 mM SO_4_(NH_4_)_2_ for ammonium-fed plants and 0.25 mM Ca(NO_3_)_2_ + 0.25 mM CaSO_4_ for nitrate-fed ones. Similarly, for 1 mM N treatment we applied 0.5 mM SO_4_(NH_4_)_2_ for ammonium-fed plants and 0.5 mM Ca(NO_3_)_2_ + 0.5 CaSO_4_ for nitrate-fed ones. It should be noted that plants grown with 0.5 mM and 1 mM N were supplied with 1.804 mM and 2.054 mM of total sulfur, respectively. Similarly, for Additional file 1: Figure S1, the sulfate supplied in the nutrient solution was 1.804, 2.805 and 4.055 mM for 0.5, 2.5 and 5 mM N treatments, respectively. Plants were maintained in hydroponic culture for 18 days. The nutrient solution of the tanks was changed on days 7, 10 and 14. The pH of the solution was monitored every two days and maintained stable between 6.8 and 7.2 during the whole experiment. A total of four tanks were used per treatment, with ten plants per tank. Thus, a total of 40 plants were grown per condition.

### Harvest

Chlorophyll content and gas exchange parameters in two plants per tank were measured in the first fully expanded leaf using a SPAD-502 Meter and an open-flow portable photosynthesis system (LICOR 6400), respectively. Chlorophyll was determined in every plant and gas exchange parameters in two plants per tank. For gas exchange, a 6 cm^2^ leaf area chamber was used making the measurements at 60% of humidity and 400 ppm CO_2_. After in vivo measurements, shoots and roots were separated and individually weighed. Then, all the plants grown in the same tank were pooled, frozen in liquid nitrogen and homogenized in a Tissue Lyser (Retsch MM 400). Samples were stored at −80 °C until use.

### Element and metabolite determination

Nitrogen, carbon and sulfur content were determined by combustion of plant dry material using an elemental analyser Flash EA1112 (Thermo Fisher Scientific Inc., Waltham, MA, USA).

Individual amino acids, ammonium, sulfate, Cys and GSH were extracted from 100 mg of frozen leaf and root powder as described in Sarasketa et al. (2014) [28]. Ammonium was quantified following the phenol hypochlorite method. Sulfate was quantified by capillary electrophoresis (Agilent G1600 CE3D, Agilent Technologies, Santa Clara, CA, USA) with indirect UV detection at 350 nm and 240 nm reference according to the standard EPA Test Method 6500 (www.epa.gov). The capillary was 40 cm long with 10 μm internal diameter and the buffer used was the Agilent Life Sciences Inorganic anion buffer pH 7.7 (Ref. 8500–6797). Nitrate content was determined spectrophotometrically from aqueous extracts according to the method reported by Cataldo et al. (1975) [29].

For amino acid and GSH determination, extracts were neutralized with NaOH and after derivatization with 1 mM fluorescein isothiocyanate, amino acids were quantified by capillary electrophoresis (PA-800, Beckman Coulter Inc., USA) coupled to laser-induced fluorescence detection (argon laser at 488 nm) as previously described [30]. Cysteine and GSH content were determined from the same extracts derivatized with 5-iodoacetamide fluorescein and reduced with tributylphosphine [31].

Glucosinolates were extracted by adding 1 mL of MeOH:water (70:30) to 25 or 50 mg of frozen leaf or root powder, respectively. The mixtures were homogenized with 2 mm glass beads in a Tissue Lyser (Retsch MM 400), incubated for 15 min at 80 °C to inactivate myrosinase and then, centrifuged for 20 min at 14000 *g*. Glucosinolates were determined from supernatants by UHPLC-QTOFMS analyses using an Acquity UPLC^TM^ from Waters (Milford, MA) interfaced to a Synapt G2 QTOF from Waters with electrospray ionization as described in Glauser et al. (2012) [32]. Glucosinolates were quantified using glucoraphanin and glucobrassicin as standards.

### Protein extraction and enzyme activities

For protein quantification total soluble protein was extracted from 100 mg of frozen leaf or root powder with 1 mL of extraction buffer as described in Sarasketa et al. (2016) [6]. Protein was quantified using a Bradford-base dye-binding assay (Bio-Rad, Hercules, CA, USA) with bovine serum albumin as a standard. Every enzyme was determined with a 96-well plate spectrophotometer (BioTek Instruments). Glutamine synthetase (GS), NADH-glutamine 2-oxoglutarate aminotransferase (NADH-GOGAT), TCA anaplerotic enzymes and glutamate dehydrogenase (GDH) in its aminating sense were determined as described in Sarasketa et al. (2016) [6]. ATP sulfurylase (ATPS) activity was measured according to Lappartient and Touraine (1996) [33]. The pyrophosphate formed during the reaction was determined at 820 nm as described by Katewa and Katyare (2003) [34]. For *O*-acetylserine (thiol) lyase (OASTL) activity, the method described by Pajuelo et al. (2007) [35] was followed quantifying the Cys formed during the reaction with ninhydrin reagent at 560 nm as described by Gaitonde (1967) [36].

### RNA extraction and qPCR

RNA was extracted from 50 mg of leaves or roots with the Nucleospin RNA plant kit (Macherey-Nagel), which includes DNAse treatment, and 1 μg of RNA was retrotranscribed into cDNA (PrimeScriptTM RT; Takara Bio Inc.). The absence of contamination with genomic DNA was tested by quantitative RT-PCR in all RNA samples. Gene expression was determined from 2 μL of cDNA diluted 1:10 in a 15 μL reaction volume using SYBR Premix ExTaqTM (Takara Bio Inc.) in a Step One Plus Real Time PCR System (Applied Biosystems). The primers used for amplifying *SULTR1;1* (AJ416460) and *SULTR1;2* (AJ311388) were described in Abdallah et al. (2010) [37]. For amplifying *SULTR1;3* (XM_013896883), the primers used were forward 5′-TTGATTGATTTCTTATCCCATGC-3′ and reverse 5′-GGAACCCTTTGAGTTGTTGAA-3′; for *SULTR2;1* (NM_001315588) forward 5′-TCTTGCAAAGCTTGATCCTC -3′ and reverse 5′-CGATTGCTATCTCTCTTGATG-3′ and for *SULTR2;2* (XM_013840590) forward 5′-TTTCAAGCCATCTTTGGACTC-3′ and reverse 5′-AGCCATGAACCCAACAAGAG-3′. The PCR program was: 95 °C for 5 min, 40 cycles of 15 s at 94 °C followed by 1 min at 60 °C; finally, a melting curve was programmed (40–95 °C with one fluorescence read every 0.3 °C). Relative gene expression was calculated as the ΔCp between each gene and the average of the housekeeping genes *ACTIN* (AF111812) with the primers forward 5′-GATTCCGTTGCCCTGAAGTA-3′ and reverse 5′-GCGACCACCTTGATCTTCAT-3′ and *EF1-a (*DQ312264) with the primers forward 5′- GCCTGGTATGGTTGTGACCT-3′ and reverse 5′ GAAGTTAGCAGCACCCTTGG-3′. The stability of the reference genes across samples was tested using geNorm software [38]. The efficiency for every primer couple was calculated with serial cDNA dilutions. The efficiencies were 2.1 for *SULTR1;1,* 1.9 for *SULTR1;2,* 2.1 for *SULTR1;3,* 2.0 for *SULTR2;1* and 2.0 for *SULTR2;2*. Data are presented respect to the highest value obtained within each tissue (value “1”).

### Statistical analysis

All the data presented are given as means with standard errors. Data were analyzed using SPSS 17.0 (Chicago, IL, USA). Statistical analysis of normality and homogeneity of variance were analyzed by Kolmogorov-Smirnov and Levene tests. The significance of the results was assessed using independent samples *t-test* and by ANOVA analysis followed by Duncan’s post hoc test.

## Results

**Fig. 1:**
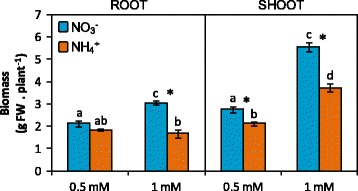
Impact of N source on *B. napus* root and shoot biomass. Plants were hydroponically cultured with 0.5 and 1 mM nitrate or ammonium. Values represent mean ± SE (*n* = 40 individual plants). Different letters indicate statistical differences between treatments (ANOVA analysis with Duncan’s test, *P* < 0.05). Asterisk (*) indicates significant nitrogen source effect within each nitrogen concentration (t-test, P < 0.05)

Accessing to a different N source may greatly influence plant performance and metabolism. In general, ammonium nutrition is considered as a stressful situation; however, there exist great inter- and intraspecific variability for the appearing of ammonium toxicity symptoms. To evaluate the performance of *Brassica napus* cv. ES Neptune under ammonium nutrition, we first screened several NH_4_^+^ concentrations in the nutrient solution of hydroponically grown plants and observed high sensitivity from 2.5 mM concentration (Additional file 1: Figure S1). Since our aim was to compare leaf and root metabolism under ammonium or nitrate nutrition before the appearance of severe ammonium stress symptoms, we chose 0.5 and 1 mM concentrations (A0.5 and A1 for ammonium-fed plants and N0.5 and N1 for nitrate-fed ones). Biomass of ammonium-fed plants was lower than that of nitrate-fed ones under both N doses. Shoot biomass of A0.5 plants was 20% lower than that of N0.5 plants while A1 shoot biomass was 33% lower compared to N1 plants (Fig. 1). For roots, no significant differences were found between A0.5 and N0.5 plants, while A1 roots were 45% smaller than N1 roots (Fig. 1). Overall, toxicity symptoms were more acute in roots. Indeed, shoot biomass positively responded to increased ammonium supply (Fig. 1, Additional file 1: Figure S1). In contrast, root biomass was similar in A1 compared to A0.5 (Fig. 1) or even decreased when increasing ammonium concentration (Additional file 1: Figure S1). Water content did not vary between treatments; it was around 90% in roots and 82% in leaves (data not shown). Although with ammonium supply growth was partially impaired, the overall plant performance was not severely altered as photosynthesis and gas exchange parameters were similar to nitrate-fed plants (Table 1). Furthermore, chlorophyll content increased upon ammonium nutrition (Table 1). Thus, A0.5 and A1 represented a mild and a moderate ammonium stress, respectively.

**Table 1:** Impact of N source on gas exchange parameters, chlorophyll content and leaf and root C content of *B. napus*

	N 0.5	A 0.5	N 1	A 1
CO_2_ assimilation (μmol CO_2_. m^−2^. s^−1^)	13.5 ± 0.5 a	14.7 ± 0.5 a	13.2 ± 0.4 a	14.4 ± 0.7 a
Transpiration (mmol H_2_O. m^−2^. s^−1^)	1.8 ± 0.1 a	1.9 ± 0.1 a	1.7 ± 0.1 a	1.7 ± 0.2 a
Stomatal conductance (μmol air. m^−2^. s^−1^)	133.7 ± 12.6 a	145.9 ± 13.6 a	126.5 ± 12.2 a	130.9 ± 14.7 a
Ci (ppm CO_2_)	187.0 ± 10.5 a	181.3 ± 11.7 a	175.2 ± 12.1 a	161.6 ± 14.5 a
Chlorophyll content (SPAD units)	**40.9 ± 0.3 a**	**51.6 ± 0.5 b**	**42.1 ± 0.4 a**	**54.5 ± 0.6 c**
Leaf C (%)	40.2 ± 0.3a	40.5 ± 0.1 a	39.9 ± 0.4 b	41.7 ± 0.2 a
Root C (%)	40.8 ± 0.2 a	40.6 ± 0.4 a	41.2 ± 0.5 a	40.6 ± 0.3 a

Plants were grown with 0.5 mM or 1 mM nitrate (N0.5, N1) or ammonium (A0.5, A1) as N source. Values represent mean ± SE (*n* = 4 for C, *n* = 8 for gas exchange parameters and n = 40 for chlorophyll). Different letters indicate statistical differences between treatments (ANOVA analysis with Duncan’s multiple comparison test, *P* < 0.05). Significant nitrogen source effect within each dose (t-test, *P *< 0.05) is highligted in bold

**Fig. 2:**
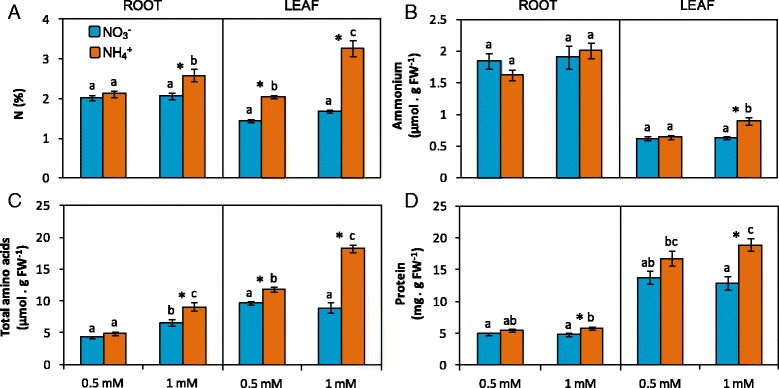
N source effect on total nitrogen (**a**), ammonium (**b**), amino acid (**c**) and soluble protein (**d**) content in roots and leaves of *B. napus*. Plants were hydroponically cultured with 0.5 and 1 mM nitrate or ammonium. Values represent mean ± SE (*n* = 4). Different letters indicate statistical differences between treatments (ANOVA analysis with Duncan’s test, *P* < 0.05). Asterisk (*) indicates significant nitrogen source effect within each nitrogen concentration (t-test, *P* < 0.05)

**Fig. 3:**
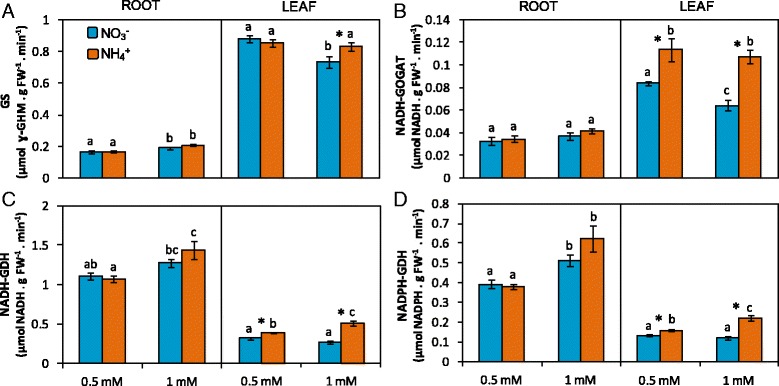
Nitrogen source effect on ammonium assimilatory enzyme activities in roots and leaves of *B. napus.* GS (**a**), NADH-GOGAT (**b**), NAD(H)-GDH (**c**) and NADP(H)-GDH (**d**). Plants were hydroponically cultured with 0.5 and 1 mM nitrate or ammonium. Values represent mean ± SE (*n* = 4). Different letters indicate statistical differences between treatments (ANOVA analysis with Duncan’s test, *P* < 0.05). Asterisk (*) indicates significant nitrogen source effect within each nitrogen concentration (t-test, *P* < 0.05)

Ammonium nutrition is known to provoke an impact on N metabolism due to NH_4_^+^ accumulation and to changes in cell metabolism coupled to the lack of need to reduce NO_3_^−^. In this work, ammonium-fed plants had higher leaf total N content both in A0.5 and A1 but only roots of A1 showed this increase in total N content compared to their nitrate counterparts (Fig. 2a). This was in agreement with both free amino acids and protein levels, which followed a parallel trend (Fig. 2c and d). In contrast, NH_4_^+^ content in tissues, which is a classical symptom of ammonium stress, did not significantly vary in roots and was only around 25% higher in A1 leaves compared to N1 (Fig. 2b). Nitrate content was very low and no differences were found between treatments (Additional file 2: Figure S2). Regarding individual amino acids, Ser and Glu were the most abundant amino acids in leaves and represented around 25% and 25–30% of the total amino acid content respectively, followed by Gln and Asp (Table 2). In roots, total amino acid content was lower than in leaves and there was no clear predominance of any individual amino acid; however, Cys, Gln, Glu, Ser, Thr, and GABA were the most abundant and each one accounted for 8 to 12% of the total amino acids content (Table 2). Both glutamine synthetase (GS) and NADH-glutamine 2-oxoglutarate aminotransferase (NADH-GOGAT) activities were higher in leaves compared to roots, in contrast to NAD(H)- and NADP(H)-dependent glutamate dehydrogenase (NAD(H)-GDH and NADP(H)-GDH), which were higher in roots (Fig. 3). Regarding N source effect, ammonium-fed plants had higher NADH-GOGAT and GS activities in leaves, although the effect on GS was slight and only significant at 1 mM concentration (Fig. 3a, b). Similarly, NAD(H)-GDH and NADP(H)-GDH activities were induced in A0.5 and A1 leaves compared to N0.5 and N1, respectively (Fig. 3c, d).

**Table 2:** N source effect on individual amino acid content (μmol . g FW^−1^) of roots and leaves of *B. napus*

	Root				Leaf			
	N0.5	A0.5	N1	A1	N0.5	A0.5	N1	A1
Ala	**0.07 ± 0.02 a**	**0.13 ± 0.04 ab**	**0.23 ± 0.04 b**	**0.54 ± 0.04 c**	**0.37 ± 0.03 a**	**0.56 ± 0.03 b**	**0.37 ± 0.03 a**	**0.56 ± 0.03 b**
Arg	0.12 ± 0.01 a	0.14 ± 0.02 ab	0.18 ± 0.02 b	0.15 ± 0.01 ab	**0.05 ± 0.00 a**	**0.07 ± 0.00 a**	0.06 ± 0.00 a	0.17 ± 0.05 b
Asn	**0.33 ± 0.02 a**	**0.20 ± 0.00 a**	0.63 ± 0.04 b	0.60 ± 0.08 b	0.13 ± 0.02 ab	0.14 ± 0.01 ab	0.08 ± 0.01 a	0.22 ± 0.06 b
Asp	0.30 ± 0.01 a	0.29 ± 0.05 a	0.25 ± 0.04 a	0.37 ± 0.04 a	1.21 ± 0.12 a	1.16 ± 0.04 a	**1.11 ± 0.18 a**	**2.07 ± 0.31 b**
Cys	0.51 ± 0.03 a	0.47 ± 0.05 a	1.01 ± 0.12 b	0.91 ± 0.08 b	0.21 ± 0.02 a	0.21 ± 0.03 a	0.18 ± 0.03 a	0.26 ± 0.03 a
Gln	**0.32 ± 0.04 a**	**0.62 ± 0.07 b**	**0.49 ± 0.05 ab**	**1.65 ± 0.11 c**	**0.46 ± 0.04 ab**	**0.91 ± 0.06 b**	**0.43 ± 0.05 a**	**2.01 ± 0.28 c**
Glu	0.52 ± 0.02 a	0.49 ± 0.03 a	**0.47 ± 0.04 a**	**0.72 ± 0.02 b**	2.87 ± 0.21 ab	3.07 ± 0.21 a	**3.10 ± 0.21 a**	**5.19 ± 0.20 c**
Gly	0.18 ± 0.02 a	0.17 ± 0.01 a	0.21 ± 0.02 a	0.24 ± 0.03 a	0.05 ± 0.01 ab	0.04 ± 0.00 a	**0.03 ± 0.00 a**	**0.05 ± 0.01 b**
His	**0.03 ± 0.00 a**	**0.02 ± 0.00 a**	0.06 ± 0.00 b	0.05 ± 0.01 b	**0.02 ± 0.00 a**	**0.05 ± 0.00 ab**	0.02 ± 0.00 ab	0.07 ± 0.03 b
Ile	**0.12 ± 0.00 a**	**0.14 ± 0.00 ab**	0.16 ± 0.02 b	0.20 ± 0.02 b	**0.06 ± 0.00 a**	**0.09 ± 0.00 b**	**0.07 ± 0.01 a**	**0.11 ± 0.01 c**
Leu	0.13 ± 0.01 a	0.14 ± 0.01 ab	0.18 ± 0.02 bc	0.19 ± 0.02 c	**0.03 ± 0.00 a**	**0.06 ± 0.01 b**	**0.05 ± 0.00 b**	**0.09 ± 0.01 c**
Lys	**0.09 ± 0.00 b**	**0.11 ± 0.01 b**	0.05 ± 0.01 a	0.05 ± 0.01 a	0.08 ± 0.02 a	0.10 ± 0.03 a	0.11 ± 0.02 a	0.14 ± 0.02 a
Met	0.02 ± 0.00 a	0.02 ± 0.00 a	0.03 ± 0.00 b	0.03 ± 0.00 b	0.04 ± 0.00 a	0.05 ± 0.00 a	0.04 ± 0.00 a	0.05 ± 0.00 a
Phe	0.06 ± 0.00 a	0.06 ± 0.01 a	0.07 ± 0.01 a	0.08 ± 0.01 a	**0.05 ± 0.01 a**	**0.09 ± 0.01 b**	**0.06 ± 0.00 ab**	**0.13 ± 0.02 c**
Pro	**0.07 ± 0.00 a**	**0.09 ± 0.00 ab**	0.09 ± 0.01 ab	0.10 ± 0.01 b	0.36 ± 0.02 a	0.44 ± 0.02 a	**0.39 ± 0.02 a**	**1.01 ± 0.08 b**
Ser	0.33 ± 0.02 a	0.35 ± 0.01 a	**0.56 ± 0.03 b**	**0.90 ± 0.03 c**	**2.81 ± 0.11 ab**	**3.29 ± 0.07 b**	**2.13 ± 0.23 a**	**4.39 ± 0.42 c**
Thr	0.38 ± 0.01 a	0.42 ± 0.02 a	0.87 ± 0.06 b	0.95 ± 0.08 b	**0.53 ± 0.04 a**	**0.91 ± 0.09 b**	**0.40 ± 0.07 a**	**0.95 ± 0.15 b**
Trp	0.07 ± 0.01 a	0.08 ± 0.01 a	**0.09 ± 0.01 a**	**0.17 ± 0.02 b**	0.15 ± 0.05 a	0.16 ± 0.05 a	**0.12 ± 0.01 a**	**0.39 ± 0.04 b**
Tyr	0.05 ± 0.00 a	0.06 ± 0.00 a	0.07 ± 0.01 a	0.07 ± 0.01 a	**0.05 ± 0.01 a**	**0.08 ± 0.00 b**	**0.06 ± 0.00 a**	**0.11 ± 0.01 c**
Val	0.26 ± 0.01 a	0.28 ± 0.01 ab	0.36 ± 0.04 bc	0.42 ± 0.04 c	**0.23 ± 0.01 a**	**0.37 ± 0.01 c**	0.22 ± 0.02 a	0.30 ± 0.03 b
GABA	0.50 ± 0.04 a	0.57 ± 0.05 ab	0.80 ± 0.11 bc	0.95 ± 0.11c	0.03 ± 0.01 a	0.04 ± 0.00 a	**0.04 ± 0.01 a**	**0.09 ± 0.01 b**

Plants were grown with 0.5 mM or 1 mM nitrate (N0.5, N1) or ammonium (A0.5, A1) as N source. Values represent mean ± SE (*n* = 4). Different letters indicate statistical differences between treatments (ANOVA analysis with Duncan’s multiple comparison test, *P* < 0.05). Significant nitrogen source effect within each dose (t-test, *p* < 0.05) is highlighted in bold

**Fig. 4:**
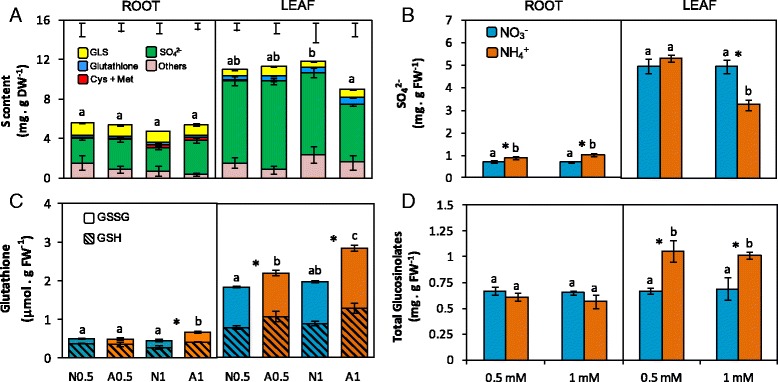
N source effect on main S-containing metabolites in roots and leaves of *B. napus.* Total sulfur (**a**) SO_4_^−2^ (**b**), glutathione (GSH and GSSG) (**c**) and glucosinolate (**d**) content. Plants were cultured with 0.5 and 1 mM nitrate (N0.5; N1) or ammonium (A0.5; A1). In panel A, different colours represent the proportion of sulfur in different compounds. Sulfur content from “other S-containing compounds” was calculated by subtracting the sum of S content in SO_4_^−2^, GSL, amino acids and glutathione from the total S concentration determined using an Elemental Analyser. Error bars at the top of the figure represent the SE of total S content. Values represent mean ± SE (*n* = 4). Different letters indicate statistical differences between treatments (ANOVA analysis with Duncan’s test, *P* < 0.05). Asterisk (*) indicates significant nitrogen source effect within each nitrogen concentration (t-test, *P* < 0.05)

Optimal S requirements strongly vary between species. The species belonging to the Brassicaceae family are among those demanding more S and this demand is associated to the elevated SO_4_^2−^ accumulation in their tissues [39]. Indeed, most of the SO_4_^2−^ taken up by cruciferous species is not assimilated but accumulated, mainly in the vacuole, and may be >80% of the total S content [40, 41]. In our work, total S content in leaves was around 1% of leaf dry weight and was twice that of roots independently of the treatment; besides, no significant N source effect was found regardless of the organ (Fig. 4a). As expected, sulfate content represented by far the highest contribution to total S content both in roots (40–57%) and leaves (59–75%). Sulfate content was slightly higher in ammonium-fed roots compared to nitrate-fed ones (Fig. 4b). In contrast, in leaves of A1 an important diminution of SO_4_^2−^ content was found compared to N1 (Fig. 4b). Indeed, this lower SO_4_^2−^ content observed in A1 leaves made that, although not significant (*t-test*; *p* = 0.094), total S was 20% lower compared to N1 leaves (Fig. 4a). Met, Cys, GSH and GLS are the most abundant S-containing organic molecules in *Brassica* crops. Met and Cys content did not vary with the N source (Table 2). In contrast, total glutathione content increased in roots of A1 and in leaves of A0.5 and A1 plants compared to their respective nitrate controls (Fig. 4c). Similarly, GLS content also increased in leaves of A0.5 and A1 plants (Fig. 4d). Eleven aliphatic, four indolic and one aromatic GLS were detected, which is in accordance with previous studies done in *B. napus* [42]. In general, the abundance of the different classes of GLS was dependent on the organ. In leaves, aliphatic GLS contributed to more than 80% of total GLS content while in roots they approximately represented 40 to 50% of the total content (Table 3). Every GLS was found in both organs except glucoiberverin and glucoerucin, which were only detected in roots (Table 3). Nevertheless, the relative content of each GLS was greatly dependent on the organ. For example, in leaves the most abundant GLS were glucobrassicanapin and progoitrin, while in roots the most abundant ones were gluconasturtin and glucoerucin (Table 3). The aromatic gluconasturtin alone represented around 40% of the total root GLS content while in leaves it did not exceed 10% (Table 3). The increase in total GLS content observed in ammonium-fed leaves compared to nitrate-fed ones was mainly due to the contribution of aliphatic GLS; among them, glucobrassicanapin and progoitrin had a major contribution (Table 3).

**Table 3:** N source effect on individual glucosinolate content (μg . g FW^−1^) of roots and leaves of *B. napus*

		Root				Leaf			
		N0.5	A0.5	N1	A1	N0.5	A0.5	N1	A1
Aliphatic glucosinolates									
Glucoraphanin	4MSOB	5.9 ± 1.1 a	6.4 ± 0.9 a	4.8 ± 0.3 a	4.9 ± 1.2 a	5.7 ± 0.6 a	5.6 ± 0.8 a	4.0 ± 0.4 ab	3.6 ± 0.3 b
Glucoalyssin	5MSOP	3.9 ± 0.1 a	3.7 ± 0.5 a	3.9 ± 0.5 a	4.2 ± 0.8 a	**37.8 ± 3.7 a**	**74.7 ± 6.6 b**	**41.3 ± 9.8 a**	**78.3 ± 5.0 b**
Glucoiberverin	3MTP	0.4 ± 0.1 a	0.3 ± 0.1 a	0.4 ± 0.0 a	0.3 ± 0.0 a	n.d.	n.d.	n.d.	n.d.
Glucoerucin	4MTB	129.8 ± 18.9 a	129.1 ± 12.6 a	114.7 ± 5.4 a	86.7 ± 11.5 a	n.d.	n.d.	n.d.	n.d.
Glucoberteroin	5MTP	70.3 ± 3.3 a	61.4 ± 6.7 bc	67.6 ± 3.2 bc	52.9 ± 5.3 c	**0.8 ± 0.2 a**	**1.7 ± 0.2 b**	**0.6 ± 0.1 a**	**1.5 ± 0.2 b**
Gluconapoleiferin	2H4P	10.9 ± 0.7 a	9.6 ± 0.8 a	**9.4 ± 0.4 a**	**6.7 ± 0.9 b**	**52.7 ± 3.4 a**	**79.7 ± 8.0 b**	47.0 ± 7.8 a	49.6 ± 8.3 a
Progoitrin	2OHB	**82.7 ± 5.1 a**	**59.0 ± 4.3 b**	64.8 ± 2.2 ab	52.1 ± 9.7 b	186.9 ± 11.7 ab	242.5 ± 25.6 b	**131.1 ± 20.2 a**	**202.9 ± 11.6 b**
Gluconapin	3B	1.2 ± 0.2 a	0.7 ± 0.1 a	2.0 ± 0.5 a	1.6 ± 0.7 a	**68.3 ± 7.3 a**	**115.3 ± 2.1 b**	84.9 ± 15.1 ab	118.8 ± 13.3 b
Glucobrassicanapin	4P	8.4 ± 1.1 a	6.0 ± 0.7 a	9.8 ± 1.5 a	8.4 ± 2.7 a	**222.4 ± 14.1 a**	**425.6 ± 40.0 b**	**202.0 ± 17.0 a**	**390.2 ± 20.5 b**
C6-aliphatic GLS A (C_13_H_24_NO_9_S_2_)		2.8 ± 0.5 a	2.2 ± 0.3 ab	**2.8 ± 0.2 a**	**1.6 ± 0.2 b**	1.3 ± 0.1 a	1.5 ± 0.1 a	1.6 ± 0.2 a	1.4 ± 0.1 a
C6-aliphatic GLS B (C_13_H_24_NO_9_S_2_)		0.4 ± 0.1 a	0.4 ± 0.0 a	0.3 ± 0.0 a	0.4 ± 0.1 a	**0.9 ± 0.1 ab**	**1 .4 ± 0.1 c**	0.8 ± 0.2 a	1.1 ± 0.0 bc
Total Aliphatic		316.4 ± 28.2 a	278.8 ± 21.7 ab	280.4 ± 9.5 ab	219.6 ± 31.0 b	**576.6 ± 31.5 a**	**925.9 ± 88.3 b**	**513.0 ± 48.8 a**	**847.5 ± 44.8 b**
Indolic glucosinolates									
Glucobrassicin	I3M	**6.8 ± 0.9 a**	**12.6 ± 1.1 a**	12.2 ± 2.3 a	20.4 ± 2.6 b	24.9 ± 1.8 a	52.6 ± 14.1 b	**25.4 ± 1.4 a**	**62.1 ± 3.5 b**
Neoglucobrassicin	IMOI3M	33.9 ± 3.4 a	28.8 ± 1.7 a	31.8 ± 2.7 a	26.9 ± 3.8 a	**7.8 ± 0.6 a**	**14.8 ± 2.2 b**	8.8 ± 1.7 ab	14.1 ± 3.0 ab
Hydroxyglucobrassicin	4OHI3M	**1.0 ± 0.2 a**	**1.7 ± 0.1 a**	1.7 ± 0.3 a	2.7 ± 0.4 a	2.9 ± 0.2 a	5.5 ± 1.2 ab	**2.9 ± 0.2 a**	**8.6 ± 1.9 b**
Methoxyglucobrassicin	4MOI3M	16.5 ± 1.5 a	16.2 ± 1.2 a	16.9 ± 1.2 a	18.1 ± 1.3 a	2.8 ± 1.0 a	3.5 ± 0.5 a	**2.1 ± 0.3 a**	**7.8 ± 1.4 b**
Total Indolic		58.2 ± 2.0 a	59.4 ± 4.2 a	62.6 ± 5.3 a	68.2 ± 7.2 a	38.4 ± 2.8 a	76.4 ± 17.8 b	**39.2 ± 2.7 a**	**92.6 ± 6.7 b**
Aromatic glucosinolates									
Gluconasturtin	2PE	261.1 ± 28.35 a	241.6 ± 26.2 a	273.2 ± 11.3 a	243.2 ± 46.2 a	42.2 ± 2.8 a	37.2 ± 1.7 a	45.5 ± 1.2 a	39.7 ± 5.7 a
Unknown GLS (C_16_H_20_N_2_O_11_S_2_)		31.9 ± 2.2 ab	26.6 ± 2.2 a	35.1 ± 1.6 b	32.2 ± 1.1 ab	8.2 ± 0.8 a	6.8 ± 0.8 a	8.6 ± 0.9 a	7.3 ± 0.3 a

Plants were grown with 0.5 mM or 1 mM nitrate (N0.5, N1) or ammonium (A0.5, A1) as N source. Values represent mean ± SE (*n* = 4). Different letters indicate statistical differences between treatments (ANOVA analysis with Duncan’s multiple comparison test, *P* < 0.05). Significant nitrogen source effect within each dose (t-test, *p* < 0.05) is highlighted in bold

**Fig. 5:**
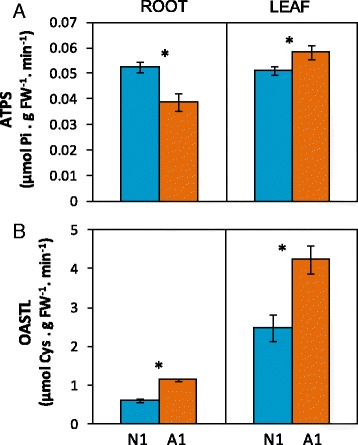
N source effect on ATPS (**a**) and OASTL (**b**) enzyme activities in roots and leaves of *B. napus.* Plants were hydroponically cultured with 1 mM nitrate or ammonium. Values represent mean ± SE (*n* = 4). Asterisk (*) indicates significant differences between nitrate and ammonium (*P* < 0.05)

**Fig. 6:**
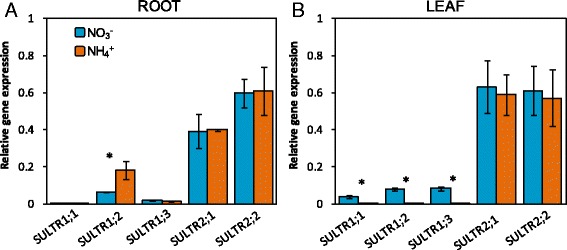
N source effect on *SULTR1* and *SULTR2* genes expression in roots (**a**) and leaves (**b**) of *B. napus.* Plants were hydroponically cultured with 1 mM nitrate or ammonium. Values represent mean ± SE (n = 4). Asterisk (*) indicates significant differences between nitrate and ammonium (*P* < 0.05)

To assess whether the substantial changes in S-compounds observed at 1 mM N concentration were related to a differential regulation of SO_4_^2−^ uptake and primary S assimilation, we checked the expression of *SULTR1* and *SULTR2* gene transporters and determined the activity of ATPS and OASTL, two key enzymes of primary S assimilation. In leaves, both ATPS and OASTL increased in A1 compared to N1 (Fig. 5). In roots, OASTL activity also increased with ammonium supply; however, ATPS activity was lower in A1 compared to N1 (Fig. 5). Besides, the expression of all the genes belonging to SULTR1 group was higher in leaves of nitrate-fed plants (N1; Fig. 6B). In contrast, in roots, *SULTR1;2* expression was higher under ammonium nutrition; interestingly, *SULTR1;2* was the member of the SULTR1 group displaying the highest expression in roots (Fig. 6a). *SULTR2* genes expression was not altered by the N source in both roots and leaves (Fig. 6).

## Discussion

### Nitrogen assimilation is enhanced in leaves of ammonium-fed *B. napus* plants

A large number of studies have associated both N availability and plant N status with the regulation of S uptake and assimilation [1, 2]. However, the studies taking into account N source effect are scarce. In this work, we studied N and S metabolism in rapeseed plants hydroponically grown with NO_3_^−^ or NH_4_^+^ as sole N source. Ammonium nutrition may be a stressful situation and its effects range from an induction in NH_4_^+^ assimilatory machinery to even plant death in function of the severity of the stress. Chlorosis is considered as a symptom of severe ammonium stress while chlorophyll accumulation has been reported as a plant response upon mild ammonium stress [43, 44]. In this work, we aimed to study N source effect avoiding severe ammonium toxicity and therefore we chose 0.5 and 1 mM N concentrations, conditions where photosynthesis or chlorophyll content remained unaffected (Table 1). Still, it was evident observing plant growth, a higher severity of the stress in A1 compared to A0.5 (Fig. 1). This situation was confirmed with NH_4_^+^ content, considered as a classical metabolic marker of ammonium stress in tissues, as only in A1 leaves increased compared to N1 leaves (Fig. 2b) while no differences were observed at 0.5 mM. Taken together, we can assume that oilseed rape plants were exposed to mild and moderate ammonium stress conditions in A0.5 an A1, respectively.

Scavenging excessive NH_4_^+^ into N-containing molecules is considered as a strategy to prevent ammonium toxicity [4, 5] and this strategy seems essential to prevent unrestrained NH_4_^+^ accumulation in *B. napus* (Fig. 2b). Indeed, NH_4_^+^ assimilation was clearly enhanced in ammonium-fed plants and notably in A1 leaves, which was reflected by higher total N, free amino acid and protein content compared to N1 leaves (Fig. 2a–d). In agreement with the higher content of N-compounds observed in ammonium-fed plants, GS and NADH-GOGAT activities increased under ammonium nutrition (Fig. 3a, b). The induction of GS/GOGAT has been commonly reported in plants grown with NH_4_^+^ as N source, including *Arabidopsis thaliana* [6, 45]. Moreover, the use of Arabidopsis mutants has revealed the importance of these enzymes in NH_4_^+^scavenging [5, 45]. NAD(H)-GDH activity or gene expression induction is also a classical response of plants exposed to ammonium stress including species of the Brassicaceae family [6, 46] (Fig. 3c). NADP(H)-GDH enzyme has been poorly characterized in plants. It is interesting that this enzyme is also induced in leaves of ammonium-fed rapeseed plants (Fig. 3d). Indeed, the induction of both NAD(H)- and NADP(H)-dependent GDH was also reported in other situations where internal NH_4_^+^ accumulated, such as in *Lotus japonicus* plants deficient in plastidic GS under active photorespiratory conditions [47]. Although studied for long time, the role of GDH in plant metabolism remains unresolved; some works indicate an exclusive GDH role for 2-OG provision [48] while in other reports an aminating role is suggested [49]. Under ammonium stress, it is somehow logic to envision an aminating function that could contribute to avoid NH_4_^+^ rise to toxic levels. However, 2-OG provision for amino acid synthesis is also crucial and thus, GDH could be working deaminating Glu. It will be interesting to study in the future whether NAD(H)- and NADP(H)-dependent GDH enzymes have a differential function in plant metabolism in general and under ammonium stress in particular. In line with the role of GDH to maintain organic acids pool, anaplerotic Tricarboxylic acid cycle (TCA) enzymes have also been suggested as important to favor NH_4_^+^ assimilation [6, 50]. In the present work, an induction of TCA enzymes was also observed in *B. napus* plants grown under ammonium nutrition (Additional file 3: Figure S3). Overall, it seems that the growth impairment observed under ammonium nutrition (Fig. 1) is not due to NH_4_^+^ accumulation in tissues but rather to the energy cost associated to maintaining NH_4_^+^ homeostasis by increasing its assimilation.

### Sulfur metabolism is induced in leaves of *B. napus* plants grown under ammonium nutrition

Sulfur uptake and assimilation are strongly interconnected with N and C metabolism [51]. In Brassicaceae, glutathione (GSH) and glucosinolates (GLS) are the major S-containing biomolecules. In leaves of NH_4_^+^-fed rapeseed plants the increase in both total GLS and total glutathione pool (GSH + GSSG) indicates that sulfur metabolism was enhanced (Fig. 4c, d). Both types of molecules are derived from amino acids, GSH from Cys, Glu and Gly and GLS mainly from Trp, Met and Phe. Indeed, we observed a general increase in free amino acid content (Fig. 2c), including Glu, Gly, Trp and Phe, in A1 leaves, together with Ser (Table 2), precursor of Cys and Met; thus, suggesting an increase in the flux of these amino acids towards GSH and GLS synthesis. According to this, ATPS activity, which catalyzes the first step of primary SO_4_^−2^ assimilation, and OASTL activity, which catalyzes the synthesis of Cys and, therefore, is the starting point for the synthesis of organic S-compounds, were induced in leaves of A1 plants (Fig. 5). Several works have also reported that the increase in amino acid availability enhanced SO_4_^2−^ assimilation, for example through an increase of the incorporation of ^35^S into proteins in *Lemna minor* [12] and in roots of *A. thaliana* [1]. Similarly, Zhu et al. (2006) [52] reported the increased expression of genes encoding S-assimilating enzymes together with a higher content of Cys and GSH in leaves of ammonium-fed rice compared to nitrate-fed one. Interestingly, in oilseed rape leaves Ser was, together with Glu, the most abundant amino acid (Table 2) and Zhu et al. (2006) [52] hypothesized that Ser, substrate of OAS to form Cys, might be the factor stimulating S assimilation under ammonium nutrition. In general, the accumulation of N in leaves under ammonium nutrition led to an increase in the availability of amino acids that was probably behind the stimulation of S incorporation into biomolecules. On the other hand, the activation of GLS and GSH synthesis could also be part of the plant strategy to store N when cell NH_4_^+^ availability is excessive. These results obtained in oilseed rape are in line with those reported in a recent study from our research group where we showed an increase in the synthesis of aliphatic and indolic GLS in shoots of *A. thaliana* and broccoli under mild ammonium stress [27]. Similarly, Zaghdoud et al. (2016) [26] also observed higher levels of indolic GLS in broccoli plants under high ammonium stress associated to Trp accumulation. Another study focused on NO_3_^−^/NH_4_^+^ ratio effect on Chinese kale, showed that the presence of NH_4_^+^ favored GLS synthesis [25]. In contrast, other works reported no changes or even decrease in GLS content when cultured with NH_4_^+^ as sole N source [23, 24]. Therefore, it seems that GLS content is dependent on the culture conditions, the *Brassica* species/genotype and the degree of ammonium stress.

The increase in GLS level has also been observed in response to other environmental factors such as salinity [53] and water deficit [54]. For instance, Martínez-Ballesta et al. (2015) [55] reported a higher impact of salt stress in Arabidopsis mutants which had lost the capacity to synthesize aliphatic GLS. Similarly, the perturbation of aliphatic GLS synthesis by RNA interference (RNAi) caused a deregulation in different metabolites and proteins related to oxidative stress and photosynthesis [56]. Therefore, we could speculate that the increase in GLS level under ammonium nutrition, a part of being a consequence of the high availability of N-reduced compounds, could be a plant response upon abiotic stress. However, the molecular mechanism by which GLS metabolism is involved in the response of Brassicaceae family to abiotic stresses still needs to be deciphered.

Interestingly, in A1 leaves the stimulation of S assimilation was accompanied by SO_4_^2−^ content diminution. However, this diminution cannot be fully explained by its recruitment into organic compounds. Sulfur is mainly taken up from the soil by SULTR SO_4_^2−^ transporters. Arabidopsis encodes 12 SULTRs which are distributed in four groups (SULTR1, SULTR2, SULTR3 and SULTR4) [57]. In this work, we examined the expression of genes from SULTR1 and SULTR2 groups, located in the plasma membrane, to try to understand the variation in SO_4_^2−^ content observed in function of the N source mainly at 1 mM concentration (Fig. 6). In roots *SULTR1;2*, the SULTR1 member with the highest expression, was induced in ammonium-fed plants (Fig. 6a), which is in agreement with the higher SO_4_^2−^ content observed in this organ (Fig. 4b). Interestingly, in A1 leaves the down-regulation of the three types of SULTR1 transporters (Fig. 5b) is in line with the lesser SO_4_^2−^accumulation in this organ (Fig. 4b). However, the accumulation of S-compounds in leaves evidences that SO_4_^2−^ availability would not be limiting probably due to the high SO_4_^2−^ content characteristic of cruciferous plants. The gene expression of SULTR1 transporters is commonly induced upon S deprivation [37, 40]; however, this induction is circumvented with the external supply of reduced S [40]. Indeed, SO_4_^2−^ uptake is commonly repressed when reduced S is available, as for instance with the exogenous supply of GSH [33, 58] or H_2_S [59]. Moreover, the inhibition of SO_4_^2−^ uptake upon H_2_S supply has even been observed in S-deficient plants [59]. Thus, in our study, the lower expression of *SULTR1* transporters in A1 leaves seems to be related to the accumulation of sulfur-containing compounds rather than to the lower SO_4_^2−^ accumulation. The overall data suggest a direct control of the N source on SO_4_^2−^ uptake/transport by SULTR1 in leaves or most probably an indirect control by a negative feedback loop triggered by the accumulation of reduced S-compounds in line with the hypothesis of demand-driven regulation of S metabolism [33, 51].

## Conclusions

The present work underlines the importance of the N source in the control of *B. napus* N and S metabolism*.* Notably, N and S assimilation was enhanced in leaves of plants grown under ammonium nutrition. The overall data highlight N source management, such as with the use of nitrification inhibitors that maintain ammonium available in the soil for long periods, as a way to stimulate S metabolism to promote the synthesis of specific molecules such as glucosinolates, which are related to plants nutritional quality in oilseed rape and cruciferous crops in general.

## Additional files

Additional file 1: Figure S1. Biomass of *B. napus* plants cultured with 1, 2.5 and 5 mM nitrate or ammonium. Values represent mean ± SE (*n* = 16 individual plants). Different letters indicate statistical differences between treatments (ANOVA analysis with Duncan’s test, *P* < 0.05). Asterisk (*) indicates significant nitrogen source effect within each nitrogen concentration (t-test, *P* < 0.05). (PDF 57 kb)

Additional file 2: Figure S2. N source effect on nitrate content in roots and leaves of *B. napus*. Values represent mean ± SE (*n* = 4 individual plants). Different letters indicate statistical differences between treatments (ANOVA analysis with Duncan’s test, *P* < 0.05). (PDF 50 kb)

Additional file 3: Figure S3. N source effect on TCA anaplerotic enzymatic activities in roots and leaves of *B. napus.* Plants were hydroponically cultured with 0.5 and 1 mM nitrate or ammonium. Values represent mean ± SE (n = 4). Different letters indicate statistical differences between treatments (ANOVA analysis with Duncan’s test, *P* < 0.05). Asterisk (*) indicates significant nitrogen source effect within each nitrogen concentration (t-test, P < 0.05). (PDF 71 kb)
