# Supplementary figures and images for: Leaves play a central role in the adaptation of nitrogen and sulfur metabolism to ammonium nutrition in oilseed rape (Brassica napus)
Source: BMC Plant Biol. 2017 Sep 20;17:157. doi: 10.1186/s12870-017-1100-9 (PMC5607504; doi:10.1186/s12870-017-1100-9)

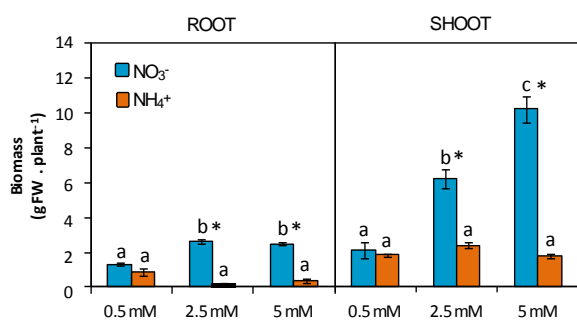

Supplement: Supplementary file 1 — Biomass of B. napus plants cultured with 1, 2.5 and 5 mM nitrate or ammonium. Values represent mean ± SE (n = 16 individual plants). Different letters indicate statistical differences between treatments (ANOVA analysis with Duncan’s test, P < 0.05). Asterisk (*) indicates significant nitrogen source effect within each nitrogen concentration (t-test, P < 0.05). (PDF 57 kb) [file 12870_2017_1100_MOESM1_ESM.pdf]

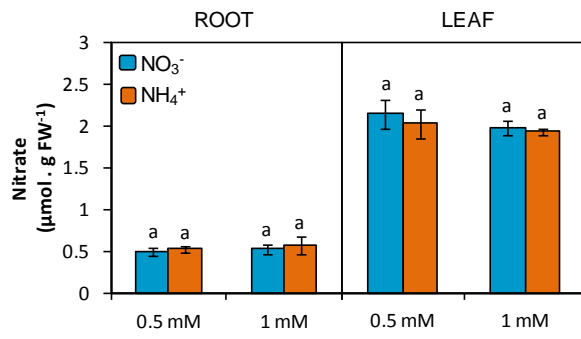

Supplement: Supplementary file 2 — N source effect on nitrate content in roots and leaves of B. napus. Values represent mean ± SE (n = 4 individual plants). Different letters indicate statistical differences between treatments (ANOVA analysis with Duncan’s test, P < 0.05). (PDF 50 kb) [file 12870_2017_1100_MOESM2_ESM.pdf]

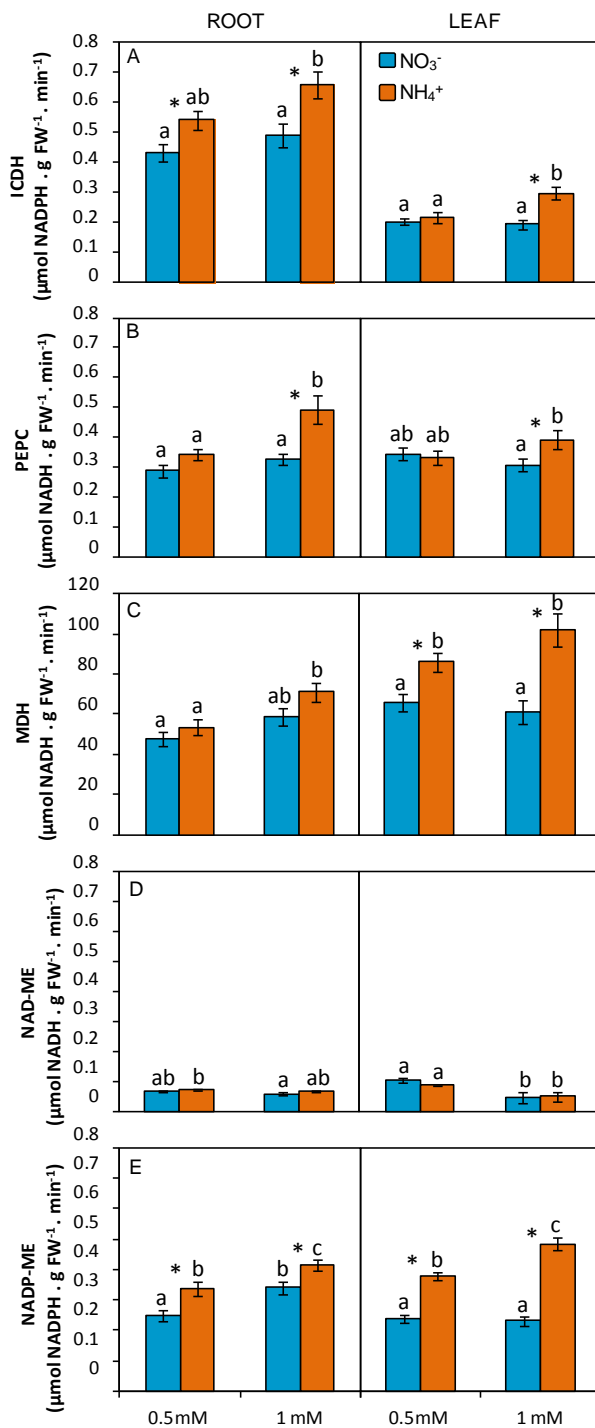

Supplement: Supplementary file 3 — N source effect on TCA anaplerotic enzymatic activities in roots and leaves of B. napus. Plants were hydroponically cultured with 0.5 and 1 mM nitrate or ammonium. Values represent mean ± SE (n = 4). Different letters indicate statistical differences between treatments (ANOVA analysis with Duncan’s test, P < 0.05). Asterisk (*) indicates significant nitrogen source effect within each nitrogen concentration (t-test, P < 0.05). (PDF 71 kb) [file 12870_2017_1100_MOESM3_ESM.pdf]
